# A Review of Animal Models of Intervertebral Disc Degeneration: Pathophysiology, Regeneration, and Translation to the Clinic

**DOI:** 10.1155/2016/5952165

**Published:** 2016-05-22

**Authors:** Chris Daly, Peter Ghosh, Graham Jenkin, David Oehme, Tony Goldschlager

**Affiliations:** ^1^The Ritchie Centre, Hudson Institute of Medical Research, Clayton, VIC 3168, Australia; ^2^Department of Neurosurgery, Monash Medical Centre, Clayton, VIC 3168, Australia; ^3^Department of Surgery, Monash University, Clayton, VIC 3168, Australia; ^4^Proteobioactives, Pty. Ltd., Balgowlah, NSW 2093, Australia; ^5^Department of Neurosurgery, St Vincent's Private Hospital, Fitzroy, VIC 3065, Australia

## Abstract

Lower back pain is the leading cause of disability worldwide. Discogenic pain secondary to intervertebral disc degeneration is a significant cause of low back pain. Disc degeneration is a complex multifactorial process. Animal models are essential to furthering understanding of the degenerative process and testing potential therapies. The adult human lumbar intervertebral disc is characterized by the loss of notochordal cells, relatively large size, essentially avascular nature, and exposure to biomechanical stresses influenced by bipedalism. Animal models are compared with regard to the above characteristics. Numerous methods of inducing disc degeneration are reported. Broadly these can be considered under the categories of spontaneous degeneration, mechanical and structural models. The purpose of such animal models is to further our understanding and, ultimately, improve treatment of disc degeneration. The role of animal models of disc degeneration in translational research leading to clinical trials of novel cellular therapies is explored.

## 1. Introduction

Lower back pain causes more global disability than any other condition worldwide [1] and is an enormous financial burden due to costs related to loss in working hours as well as for its medical treatment. Up to 80% of people may experience lower back pain at some stage in their life, with prevalence ranging from 15 to 45%. Chronic lower back pain can be caused by degenerative lumbar disc disease which produces discogenic pain [2]. This needs to be distinguished from radicular pain, which is pain resulting from nerve root compression, often due to a disc prolapse. Lumbar disc degeneration is a complex process manifested by changes in cellular, matrix, endplate, and the neurovascular components of the intervertebral disc. Given the significant contribution of disc degeneration to the enormous disease burden of lower back pain numerous animal models have been developed in an effort to further understanding and treatment of this condition. In order to compare and contrast the merits of different models a basic appreciation of the structure of the intervertebral disc and underlying pathophysiology is a prerequisite.

## 2. The Intervertebral Disc

The intervertebral disc is a complex multicomponent structural tissue consisting of an outer fibrous ring, the annulus fibrosus (AF), and an inner hydrated gel-like substance, the nucleus pulposus (NP) [3]. It is the largest avascular structure in the body. Nutrition of the intervertebral disc is provided by diffusion through the cartilaginous endplates (CEP). The CEP are specialized interfaces that connect the intervertebral disc with the adjacent vertebral bodies. The AF is a fibrocartilaginous tissue rich in type I and II collagen and assembled as lamellae fibres oriented at varying degrees to adjacent lamella in different locations and species. The AF connects the caudal and cranial vertebral bodies of the spinal column [4]. The main cell types of the AF are fibroblasts that synthesize not only the lamellar collagen, but also proteoglycan (PGs), elastin, and other noncollagenous proteins [5]. The tough fibrous composite structure of the AF encapsulates the gelatinous NP and provides the necessary mechanical strength and resilience to allow the disc to recover from deformation arising from axial, rotational, and bending loading. In healthy discs the NP consists of a hydrated gel composed of predominantly type II collagen and large amounts of PGs. Aggrecan is the most abundant PG type in the NP. Due to its high anionic charge aggrecan attracts and retains high levels of water molecules within the NP thereby maintaining a high hydrostatic swelling pressure that confers resistance to disc deformation and maintenance of disc height [3, 6].

Cells of the disc NP are derived from the notochord. In man these cells are retained throughout childhood but with maturity disappear and are replaced by chondrocyte-like cells [5]. The loss of notochordal cells from the NP represents an important early step along the path to degenerative disc disease and in this regard it should be noted that apart from a limited number of animal species (chondrodystrophoid dogs, old sheep, and cattle) NP notochord cells are retained throughout their life.

## 3. Intervertebral Disc Degeneration

Human intervertebral disc degeneration is a complex and incompletely understood multifactorial process with contributions from genes, mechanical stresses, cellular senescence, and alterations in nutrition via the limited vascular supply [7]. With respect to mechanically loading the intervertebral disc there is a delicate balance between “normal” mechanical loading, which is required for maintenance of an optimal disc cellular phenotype [8, 9], and excessive mechanical loading that causes damage. Excessive loading can result from excessive bodyweight [10] or trauma and produces many of the features of degeneration that can be visualized by histological and radiological methods.

Studies comparing degenerate discs with nondegenerate controls have demonstrated increased evidence of senescent cells in degenerate intervertebral discs [11]. Such cells lose the ability to divide, thus potentially contributing to the decreased cellularity of the diseased degenerate intervertebral discs. Moreover, the senescent cells have a reduced ability to function. Thus they produce less matrix which, in turn, further compromises the structure of the intervertebral discs.

Intervertebral discs comprise the largest essentially avascular tissue in the human body. Only the outermost layers of the AF contain blood vessels. The cells of NP are dependent on diffusion of nutrients from capillary buds in the cartilaginous endplate to meet their metabolic needs [12]. The cells in the NP are therefore metabolically compromised by this limited vascular and nutritional supply and may promulgate intervertebral disc degeneration. Causes of impaired nutrition to the intervertebral disc include endplate calcification, microvascular disease, and smoking and have all been associated with early disc degeneration.

**Figure 1:**
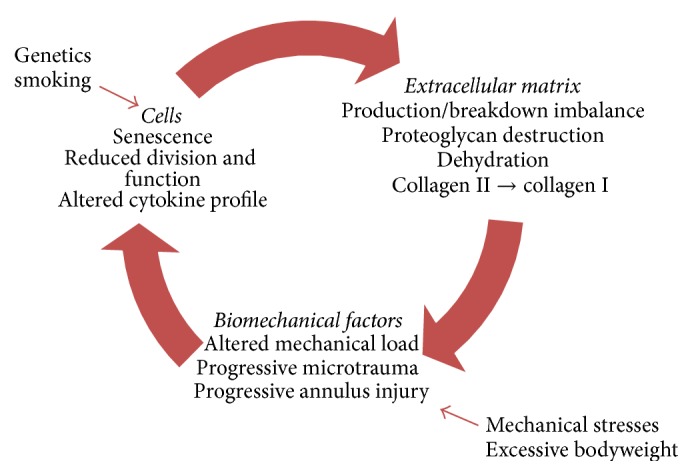
Schematic of the process of disc degeneration demonstrating multifactorial pathophysiology and interplay of cellular, matrix, and biomechanical factors. Modification of figure from Vergroesen et al. [17].

Ultimately there is an imbalance between the rates of production and breakdown of the matrix components leading to a cascade of events (see Figure 1) consisting of alterations in matrix synthesis, reduced aggrecan synthesis, and a transition from type II to type I collagen production [13], reduction in cellular viability and activity, and alterations in cytokine profile upregulating the breakdown of proteoglycans, all leading to dehydration and loss of mechanical integrity of the intervertebral disc [6, 12]. The dehydration of the intervertebral disc reduces the mechanical support provided by the swelling pressure of the previously hydrated NP. This alters the mechanical load to which the AF is exposed and thus the tension in the AF collagen fibres. This leads to subsequent progressive microtrauma of these fibres [6]. The degeneration of the AF and subsequent tears in this structure predispose patients to disc herniation, wherein fragments of disc tissue herniate through this annular defect causing neural compression and radicular pain [14]. As the mechanical and structural integrity of the disc progressively deteriorates neurovascular invasion may occur via annular tears. Neurovascular invasion extending to the NP via annular fissures has been demonstrated in painful discs in clinical studies [15]. In contrast, control (nonpainful) discs demonstrated restriction of vascular and neural supply to the outer annulus [15]. This process of neoinnervation of the degenerate intervertebral discs is hypothesized to be a significant contributor to the development of back pain [16].

### 3.1. Animal Models of Disc Degeneration

The development of appropriate animal models of intervertebral disc disease is necessary to gain insight into the pathophysiology and to develop and test potential therapies. In vitro and in silico (computer based) systems can be helpful to investigate specific components of intervertebral disc degeneration. However, given the complexity inherent in the intervertebral disc with biochemical, biomechanical, nutritional, and metabolic factors acting simultaneously, in vivo animals are able to more faithfully replicate this environment. A range of animal models and mechanisms of replicating the process of degeneration have been investigated and utilized in efforts to develop appropriate models of intervertebral disc degeneration. However, given the extreme complexity of this system no perfect model currently exists.

Initial discussion will focus on the merits and inadequacies of particular species as models for human intervertebral disc degeneration. This will be followed by a discussion of the comparative merits of the various methods of inducing disc degeneration.

#### 3.1.1. Comparison of Various Animal Models

Animal models range from small rodents such as mice knockout models [18] to rats [19], rabbits [20], dogs [21], goats [22], sheep [23], and primates [24, 25]. Various mechanisms of inducing degeneration have been described for these animal models which are summarized in Table 1. When considering the potential suitability of an animal model several important characteristics must be taken into account and these are discussed in Table 1.

#### 3.1.2. Persistence of Notochordal Cells

The vertebral column and thus intervertebral discs of all mammals arise from aggregation of the mesenchyme around the notochord and subsequent segmentation during development [26]. Notochordal cells persist in the NP of the majority of species (e.g., mice, rats, rabbits, and pigs) into adulthood. However, the number of these cells decreases rapidly following birth in humans and notochordal cells are completely absent from the NP by early adulthood [26]. Sheep and goats are among the few animals to also lose the notochordal cells rapidly from the NP following birth. Dogs are divided into two populations with regard to notochordal persistence into adulthood. Chondrodystrophoid (CD) dogs rapidly lose the notochordal cells following birth and as such are predisposed to intervertebral disc degeneration in later life. Nonchondrodystrophoid (NCD) dogs have persistent notochordal cells and are far less inclined to disc degeneration. The persistence of notochordal cells is an important consideration as these cells have a significant influence on the intervertebral disc by influencing proteoglycan metabolism [27, 28], hyaluronan production [29], and possible progenitor cell function [26].

Loss of intervertebral disc notochordal cells may be observed in animal models with otherwise persistent notochordal cells following adequate stimulus [30, 31]. Apoptotic processes have been demonstrated to play a significant role in this process of notochordal cells loss [31, 32] and are also observed in human aged and degenerate discs [33]. Thus such animal models may have greater relevance following the loss of notochordal cells than otherwise.

However, given the use of animal models to investigate cellular regenerative therapies for the treatment of disc degeneration the potential presence of a preexisting precursor cell population may complicate investigation of the regenerative potential of such therapies. For instance, in cell transplantation therapies, one cannot be sure that it is not the resident notochordal cells which are responsible for the regenerative effects, instead of, or in combination with, the transplanted cells.

#### 3.1.3. Disc Size and Geometry

Intervertebral discs vary markedly across species and according to location within the spine. The discs of most animal models are smaller than human intervertebral discs. Disc size affects solute diffusion in the intervertebral disc. Given the largely avascular nature of the intervertebral disc and dependence on diffusion to meet nutritional requirements this is of particular significance to the clinical relevance of animal models. Given the size discrepancies between common animal models and humans investigators have analyzed disc geometry hoping to better determine the relevance of particular models to the human intervertebral disc. In a study by O'Connell et al. [34] the geometries of intervertebral discs of commonly used animal models were analyzed with regard to their similarity to the human intervertebral discs as measured by relative proportions (e.g., disc height, width, and NP size). Interestingly, the authors ranked mouse lumbar intervertebral disc as the animal model most geometrically analogous to the human intervertebral disc.

#### 3.1.4. Disc Mechanical Forces

The vast majority of animal models of intervertebral disc disease are quadrupedal. The only bipedal models available, certain primates to varying extents (e.g., rhesus monkey [35]) and the bipedal mouse and rat [36], present ethical dilemmas that preclude their usage in most institutions. Given that the mechanical loading to which human intervertebral lumbar discs are exposed is significantly influenced by the upright posture it may be thought that this precludes usage of quadrupedal animal models. However, muscle contraction and ligament tension is a significant contributor to the load to which intervertebral discs are exposed [37]. It has been hypothesized that the load exerted on the lumbar intervertebral discs of large animals by these structures may be even greater than that observed in humans resulting from the bipedal stance due to the increased complexity of stabilizing a horizontally aligned spine versus a vertically balanced spine [26].

### 3.2. Animal Models

Taking the above general considerations into account the following models are those most commonly described for use as in vivo models of intervertebral disc degeneration.

#### 3.2.1. Rodent Models

Mice and rat models, despite the obvious difference in intervertebral disc size, have significant advantages with regard to ease of use and application of technology. Genetic knockout and mutation mice models have enabled the investigation of the effects of the elimination of particular proteins, for example, collagen II [18], on disc function. Bipedal mouse and rat models were created through bilateral mid-humeral surgical and tail amputations [36]. Bipedal mice were observed to demonstrate accelerated NP degeneration with frequent NP herniation [40]. However, more recent studies have indicated that bipedal rats do not assume a more erect posture than their quadrupedal peers [69] calling into question the cause of the observed increased disc degeneration.

The mouse and rat tail provide a readily accessible model for intervertebral disc degeneration through mechanical injury, asymmetrical compression, or administration of digestive enzymes [39, 70].

However significant limitations exist for such models:

1. Persistent notochordal cells: limiting the potential relevance of such models to the clinical environment particularly with regard to testing potential therapeutics.
2. Differing mechanical loading: rodent tail models: the tail may have significantly different mechanical loading to the human lumbar spine although this has been disputed by some authors.
3. Significantly smaller disc size reducing the nutritional challenge.
4. Ethical concerns regarding the bipedal mouse.

#### 3.2.2. Rabbit Models

The rabbit model of intervertebral disc degeneration has been utilized by several authors for investigation of disc degeneration and of potential therapeutic agents [20, 71]. Major advantages of this model are the higher degree of homology to the human intervertebral disc with the presence of facet joints and paravertebral muscles and ligaments in comparison to the rodent tail models [51], the larger size of the animal and intervertebral discs, and the cost-effectiveness as a model relative to large animals. Limitations relate to the persistence of notochordal cells and the significant variation from human geometry [72].

#### 3.2.3. Canine Models

As discussed previously CD dogs demonstrate a decrease of notochordal cells from birth onwards with complete loss by early adulthood predisposing the animal to intervertebral disc degeneration. The CD dogs, among which beagles and dachshunds number, are well-characterized models of spontaneous intervertebral disc degeneration [57]. The larger size of the disc space relative to rodents makes administration of intradiscal therapeutic agents technically less challenging [58]. Similarities exist with regard to the gross pathology, histopathology, and glycosaminoglycan content among humans and canines in intervertebral disc disease [21]. Differences exist with regard to the thicker cartilaginous endplates in humans, the presence of growth plates within the vertebrae of the canine [21], and intervertebral disc size. Additional ethical concerns exist with regard to the use of dogs for experimental research in many parts of the world.

#### 3.2.4. Goat Models

Goats have previously been used as models for intervertebral disc degeneration [22, 60]. Advantages of the use of this species include anatomical similarities with regard to size and shape with respect to the human intervertebral disc, comparable mechanical load [73], absence of notochordal cells in the adult [74], and the pragmatic benefits of a hardy, economical animal model that tolerates surgery well [22].

#### 3.2.5. Sheep Models

The sheep model has proven to have particular merit for several major reasons. Firstly, the sheep, similar to humans, suffers from a loss of notochordal cells in early adulthood, predisposing the sheep intervertebral discs to degeneration [75]. The sheep is of a roughly similar size to humans and, despite its quadrupedal stature, is exposed to very similar mechanical stresses to the human intervertebral disc [76]. The ovine spine has previously been used to model disc degeneration [62–64] and test implant devices and in the preclinical investigation of cellular therapies [23, 77–79]. Similar to the goat the sheep is a hardy animal with demonstrated ability to tolerate surgical intervention.

#### 3.2.6. Porcine Models

Porcine models have been utilized in models of intervertebral disc degeneration and in the preclinical assessment of biological therapies such as mesenchymal stem cells [61, 80]. Major advantages attributed to the porcine model include the similarity in size of the disc to the human intervertebral disc and overall size of the animal. However, this advantage is significantly offset by the persistence of notochordal cells into adult life in the porcine model [80], potentially confounding interpretation of investigations of disc degeneration and regenerative therapies.

#### 3.2.7. Primate Models

Spontaneous disc degeneration has been demonstrated in baboon and macaque models [24, 25, 66]. Both species are quadrupedal for locomotion but spend large amounts of time in semierect and erect positions. Rhesus monkeys have also been used as models of disc degeneration following annulotomy ± intradiscal administration of collagenase [67] and subchondral administration of bleomycin [68]. The advantages of such nonhuman primate animal models include intervertebral disc size closer to humans, comparable anatomy, spontaneous disc degeneration, and exposure to mechanical stresses compatible with erect posture. However major ethical and practical considerations (e.g., expense and housing) are associated with the use of nonhuman primate animal models significantly restricting their use for such studies in many institutions.

### 3.3. Comparison of Mechanisms

Given the complexity of human disc degeneration no animal model can perfectly mimic the entire pathophysiological process. Disc degeneration in animal models is typically initiated by various chemical (e.g., chondroitinase ABC injection [81]) or mechanical (e.g., surgical incision [63], nucleotomy-NP aspiration [82], and drill bit injury [22]) stimuli though it can develop spontaneously in some animals [83].

#### 3.3.1. Spontaneous Models

Spontaneous disc degeneration occurs in a limited number of species, with inconsistent onset and development over a long time frame. The most well-studied species with regard to spontaneous disc degeneration are the sand rat and the chondrodystrophoid dog species. As described above spontaneous disc degeneration has also been observed in nonhuman primates.

*Sand Rat.* The sand rat is native to east Mediterranean deserts [84] and was first described to undergo spontaneous disc degeneration by Silberberg et al. [48]. When fed a standard laboratory diet the sand rat develops diabetes and widespread disc degeneration [85]. The degenerative changes consist of loss of notochordal cells, annular disorganization, cellular metaplasia, endplate sclerosis, and the formation of peripheral osteophytes [50]. In a longitudinal study conducted by Gruber et al. [49] radiographic evidence of degeneration was evident by two months with older animals demonstrating disc space wedging, narrowing, irregular disc margins, and endplate calcification. The degenerative process commenced in the NP with subsequent degeneration of the facets and endplates occurring only after disc herniation had developed [48]. Additionally the sand rat has been successfully used in studies of cellular therapy of disc degeneration despite the significant technical challenges this entailed [86].

*Genetically Modified Mice.* Genetically modified mice models have been developed to investigate the contribution of specific proteins to disc degeneration. Genetically modified mice with collagen IX mutations demonstrated increased cervical degeneration [38]. Similarly mice with collagen II mutations underwent premature endplate calcification and subsequent disc degeneration [18].

*Canines.* As detailed previously CD dogs demonstrate loss of notochordal cells from birth onwards and progress to demonstrate gross pathology and histopathological and glycosaminoglycan content changes similar to humans in intervertebral disc degeneration [21]. There are also marked similarities between magnetic resonance images of intervertebral disc degeneration in different stages of progression in canines and humans [87, 88]. Canines (both CD and NCD) also undergo routine clinical treatment for degenerative disc disease including decompressive surgery [21]. The chondrodystrophoid dog has long served as an animal model of intervertebral disc degeneration and will continue to do so into the future.

*Primates.* Baboons and macaques have both been demonstrated to undergo spontaneous disc degeneration [25, 66]. As nonhuman primates that spend a significant proportion of time in erect and semierect postures such animal models demonstrate significant potential for modeling human disc degeneration. However, ethical and pragmatic consideration will likely limit their usage.

 Spontaneous models of disc disease can be particularly useful in providing models of disc degeneration. However, the long and at times unpredictable course of spontaneous degeneration often limits their utilization in studies of potential therapies.

#### 3.3.2. Mechanical Animal Models of Disc Degeneration

Mechanical models afford the advantage of initiating the degenerative cascade at a defined time point in a replicable fashion. Epidemiological studies have suggested the association between exposure of the spine to force and disc degeneration [89]. Mechanical models of disc degeneration can be broadly divided into two groups: compression and instability, although there is overlap between the two groups [83].

*Compression.* Compression involves the application of altered mechanical stresses to the intervertebral disc through mechanism such as bending [43], postural change [90] (i.e., the bipedal rat), or cyclical compression [45].

*(1) Bending.* Bending of the rat tail is one of the earliest reported methods of inducing disc degeneration [43]. In pioneering studies by Lindblom [43] rat tails fixed into bent shapes demonstrated annulus degeneration on the concave side with connective tissue injury and reduced cellularity. In a more recent study Court et al. [39] were able to demonstrate increased cell death and decreased aggrecan gene expression in the concave side of a disc compressed by forceful fixed mouse tail bending. Such differences were not observed in mice tails exposed to only slight bending.

*(2) Postural Change*. The bipedal rat and mouse models, as described above, are based on the hypothesis that surgically modified animals will spend more time in an erect posture thus exposing the intervertebral discs to increased mechanicals stress. Given the more recent findings indicating bipedal rats do not spend an increased time in an erect posture and, in fact, possibly less time than their quadrupedal counterparts [69] it is interesting to reflect as to the aetiology of the underlying degenerative changes observed in the primary studies.

*(3) Chronic and Cyclical Compression.* Researchers have investigated the application of static and cyclical compression to the intervertebral disc. Iatridis et al. [44] described a rat tail compression model to apply chronic compression. This consisted of an Ilizarov-type apparatus (an external fixation device enabling application of mechanical force across the intervertebral disc) applied to the tail of rats. Rats were assigned to sham, immobilization, or compression groups. The immobilization and compression groups demonstrated decreased disc thickness, axial compliance, and angular laxity with the compression group demonstrating these changes more quickly and with greater magnitude. Interestingly the discs demonstrated increased proteoglycan content in contrast to human disc degeneration, in which reduced proteoglycan content is observed during the degenerative process.

 Kroeber et al. [51] developed a novel model that enabled the application of compressive force to the intervertebral discs of rabbits via attachment of an external loading device. Rabbit intervertebral discs were exposed to up to 28 days of loading of a disc compressive force equivalent to five times bodyweight. After 14 and 28 days of loading discs demonstrated significantly reduced disc space with annulus disorganization observed histologically. Increased dead cells were observed in the annulus and endplate. These changes were not reversible after an equivalent period of unloading.

Cyclical compression has also been investigated. In a rat tail model Ching et al. [45] investigated the effects of static and cyclical loading at 0.5, 1.5, or 2.5 Hz. Pins were inserted into the caudal 4th and 5th vertebrae. A compression device was applied to these pins. The greatest loss of interpin distance (a measure of intervertebral disc height and thus disc degeneration) was observed in rat tails subjected to static compression with the least loss of interpin distance, other than the sham control, observed in the 1.5 Hz group, suggesting disc response varies with the frequency of loading.

*Instability.* Various animal models of disc disease exist in which the intervertebral disc is exposed to increased instability at the motion segment promoting intervertebral disc degeneration. Approaches to produce instability include surgical resection of posterior elements such as facet joint and spinous processes [41] and the fusion of an adjacent level [52]. Miyamoto et al. [41] demonstrated that resection of spinous processes, supraspinous and interspinous ligament, with paravertebral muscle detachment accelerated cervical disc degeneration in a mouse model. At 12 months following surgical intervention the experimental group demonstrated advanced disc degeneration with intervertebral disc material herniation, AF disorganization, metaplasia of fibroblastic cells in the AF into chondrocytes, loss of disc height, and osteophyte formation.

Phillips et al. [52] reported a novel method of modeling intervertebral disc degeneration in the rabbit by performing simulated surgical spinal fusion at the lumbar L5–L7 level in rabbits. Spinal fusion eliminates movement at the index level but induces altered stresses at the adjacent mobile segments [91, 92]. The adjacent level intervertebral discs L4-L5 and L7-S1 demonstrated progressive disc degeneration. Annular disorganization with loss of normal collagen bundle arrangement was observed at three months. This was increased at six months and by nine months the normal structure of the disc had been replaced by disorganized fibrous tissue, annular tears, and loss of chondrocytes and notochordal cells in the NP were observed as was decreased monomeric size of the proteoglycans. Furthermore disc space narrowing, endplate sclerosis, and osteophyte formation were also observed in keeping with the clinical condition.

Instability studies allow an inducible method of progressive disc degeneration with many of the features observed in the clinical condition. The time course of progression of these models, requiring 9–12 months for the establishment of severe disc degeneration, may preclude their usage in studies of regenerative therapies given cost concerns.

#### 3.3.3. Structural Models

An alternate mechanism of inducing disc degeneration is directly compromising the structural integrity of the intervertebral disc. This task can be accomplished by chemical or direct physical methods.

*Chemonucleolysis.* Various chemical agents have been investigated as potential stimuli to induce the pathophysiological process of disc degeneration. The best described such agent, chymopapain, was first reported in clinical use in 1964 for the treatment of sciatica secondary to presumed disc protrusion [93]. Chymopapain is a proteolytic enzyme derived from the papaya latex [94] that produces disc degeneration by inducing proteolytic digestion or removal of glycosaminoglycan chains. Proteoglycan loss leads to disc height loss and altered biomechanical stability [26]. The enzyme selectively degrades intervertebral disc proteoglycan in a dose dependent fashion [59]. Inadequate doses may be followed by NP proteoglycan restoration [95]. High doses have also been demonstrated to directly produce annulus destruction in animal models [53].

Chondroitinase ABC is another enzyme demonstrated to produce disc degeneration in animal models [96]. Chondroitinase causes its effect by producing degradation of the polysaccharide side chains of the proteoglycans of the intervertebral disc [97]. Chondroitinase ABC injection was demonstrated to produce dose dependent intervertebral disc degeneration in a caprine model by Hoogendoorn et al. [98]. Chondroitinase ABC injection has also been used in an ovine model to induce intervertebral disc degeneration to enable assessment of regenerative therapies [65].

As described above injection of enzymes leads to proteoglycan loss, an essential component of the pathophysiological process of disc degeneration observed clinically. A criticism of the chondroitinase ABC for inducing disc degeneration is that the viability of native disc cells is largely preserved, enabling regeneration of the extracellular matrix [99].

*Physical Methods.* Surgical injury to the intervertebral disc is a well-established method of inducing disc degeneration. Injury can be performed to the endplate, the annulus, or the annulus and nucleus.

Endplate injury has been demonstrated in a porcine model to produce changes consistent with disc degeneration. Following lumbar endplate injury with a drill bit porcine intervertebral discs were observed over a 3-month period to demonstrate annular delamination, with reduction in nucleus proteoglycan content, cellularity and loss of gel-like structure [100]. Evidence of degeneration of varying degrees of magnitude was observed seven months following injury in a similar porcine model of endplate injury induced disc degeneration [101].

**Figure 2:**
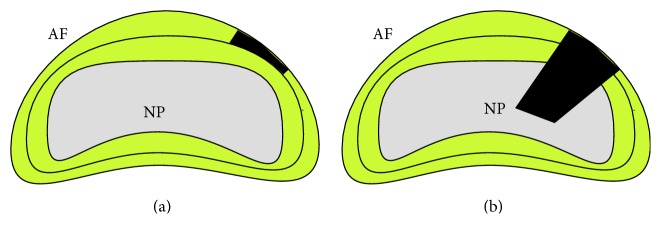
(a) Partial thickness annular injury. (b) Full thickness annular injury with NP involvement. AF indicates annulus fibrosus; NP indicates nucleus pulposus.

Annular injury models were first described in the 1930s by Keyes and Compere [55]. Keyes and Compere demonstrated that annular injury with subsequent NP expulsion leads to loss of disc height and degenerative changes at the index level. Following these pioneering studies multiple intervertebral disc injury methods have been investigated for their potential to induce disc degeneration. Broadly such methods can be considered under the categories of partial thickness annular injury and full thickness annular injury with nucleus involvement (see Figure 2). Full thickness annular injuries have the advantage of producing nuclear avulsion with relatively rapid degeneration. Partial thickness injuries produce a slower degenerative process.

Stab injuries and annulotomies have been performed in a variety of animal models including rats [46], rabbits [20], sheep [62], and pigs [61]. Osti et al. [62] demonstrated in an ovine model that partial thickness annular injury, consisting of a 5 mm depth incision that left the inner annulus and NP intact at the time of injury, would lead to progressive failure of the inner annulus with progressive disc degeneration over several months. Oehme et al. [63, 102] demonstrated in an ovine model that after three months a larger (20 mm × 6 mm) partial thickness annular injury resulted in significant increased disc height loss, increased MRI Pfirrmann degeneration scores, increased histological injury scores, and decreased NP glycosaminoglycans in the injured discs.

Full thickness intervertebral disc injury is demonstrated in the approach of Oehme et al. [23]. In this injury model a simulated partial lumbar microdiscectomy was performed by creating a 3 × 5 mm annular incision in ovine discs followed by removal of 0.2 g of intervertebral disc tissue, including NP. 24 weeks following performance of the partial-microdiscectomy injured and otherwise untreated intervertebral discs demonstrated increased disc height loss, increased MRI Pfirrmann degeneration scores, and reduced NP proteoglycan content relative to controls.

**Figure 3:**
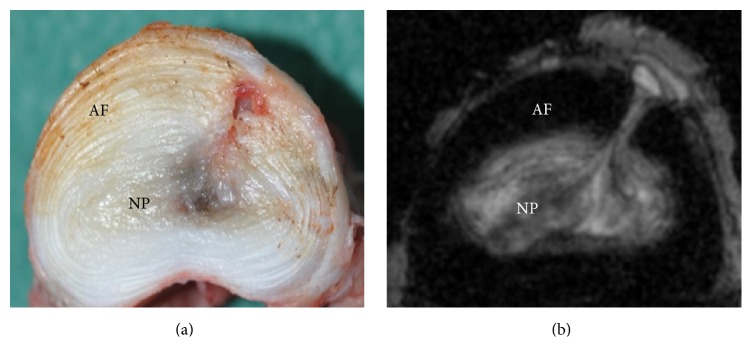
(a) Ovine drill bit injured intervertebral disc demonstrating injury penetrating through the annulus into the nucleus. (b) 9.4 T axial MRI T2 sequence demonstrating drill bit injury tract extending through AF to NP. AF indicates annulus fibrosus; NP indicates nucleus pulposus.

A novel full thickness intervertebral disc injury caprine model utilizing a drill bit has recently been described by Zhang et al. [22]. The authors compared scalpel blade annulotomy with insertion of a 4.5 mm drill bit to a depth of 15 mm. At two months the drill bit injured intervertebral discs demonstrated significantly increased histological injury scores relative to controls. This injury model has served as the stimulus for investigation in our laboratory utilizing an ovine model. The drill bit injury model has the advantage of producing a highly replicable injury demonstrated in the goat to produce disc degeneration over a two-month period. Drill bit injury was performed by insertion of a 3.5 mm drill bit 12 mm in depth in two adjacent ovine lumbar intervertebral discs. Sheep underwent necropsy at two months. Gross morphology and 9.4-tesla MRI demonstrated significantly increased injury scores in injured versus control discs (see Figure 3).

## 4. Involvement in Preclinical Trials

Despite the limitations of the animal models described above such models play an integral role in increasing our knowledge and understanding of the process of disc degeneration and in the development of novel therapies for clinical application. Given the complex pathophysiological process of disc degeneration with the interplay of cellular, biomechanical, and matrix components cellular therapy is considered by many to demonstrate the greatest potential in the treatment of this condition.

A recent review by Oehme et al. [103] comprehensively details the variety of preclinical and clinical trials of novel cell-based therapies for the treatment of lumbar intervertebral disc degeneration. Animal models used in preclinical trials of novel therapies include rat [47], rabbit [54], canine [56], porcine [61], ovine [23, 78, 79], and rhesus monkeys [104]. The vast majority of animal models described utilized full thickness annular injury with nuclear involvement to induce disc degeneration. As detailed above, the advantage of this injury model is the ability to consistently induce degeneration at a specified time point. Cell types investigated for regenerative potential include NP chondrocytes [56], bone marrow derived mesenchymal stem cells [54], and bone marrow derived mesenchymal precursor cells [23]. The three cell types detailed are notable for having demonstrated the ability to promote intervertebral disc regeneration in preclinical trials with subsequent progression to clinical trials/series.

### 4.1. Clinical Translation

The EuroDISC clinical trial [105] investigated the transplantation of expanded autologous disc chondrocytes in patients undergoing single level discectomy. Interim analysis of 28 patients at 24 months revealed those patients who received chondrocyte transplantation reported greater pain reduction and demonstrated increased disc fluid content on MRI compared to controls. Percutaneous injection of expanded autologous mesenchymal stem cells in two small noncontrolled clinical trials leads to improved MRI T2 signal and clinical improvement [106, 107].

Autologous bone marrow mesenchymal stem cell administration has been investigated in two small series of patients [106, 107]. The trial of Orozco et al. [106] reported clinical improvement in 9 of 10 patients who received expanded autologous MSCs for treatment of low back pain with degenerative disc disease and failure of conservative treatment. The series of Yoshikawa et al. [107] consisted of two patients who at two years both reported significant improvement with improved disc hydration on MRI.

The administration of allogeneic mesenchymal precursor cells for the treatment of back pain has been investigated in a Phase II study [108]. A significantly greater proportion of MPC treated patients achieved minimal residual back pain and at least a 50% reduction in back pain. Phase III trials are now underway.

### 4.2. Pain

The discussion of clinical translation raises one of the most important considerations regarding the translation of findings from animal models of disc degeneration, that of pain. Disc degeneration causes the majority of its morbidity and disability through back pain—a subjective phenomenon. Pain is a symptom experienced by patients and is multifactorial in nature. The clinical observation of significant radiological disc degeneration in the absence of significant back pain in many patients is suggestive of the notion that the two are not necessarily well correlated at all times. Thus the measures of disc degeneration employed in animal models of disc disease such as histology and radiological degeneration scores and macroscopic and biochemical analysis can serve as useful markers of underlying disc degeneration but can inform the observer to only a limited degree of the likely disability associated with such findings.

The assessment of intervertebral disc degeneration related pain in animal models is still in its relative infancy. The majority of such research has been conducted in rodent models [109, 110]. Pain in rodents can be assessed in three ways [110]: observation of pain-related behaviours (e.g., increased grooming and “wet-dog shakes” [111]), measuring functional performance (e.g., locomotor ability assessment in mice [109]), or determining response to mechanical or thermal stimuli (e.g., grip strength in response to axial stretch, a possible measure of axial low back pain). A recent study comparing sensitivities of different pain assessment methods in a rat model suggested hind paw mechanical sensitivity and duration of grooming as the most sensitive measures of degeneration induced pain [110]. Hind paw mechanical sensitivity offers the advantage of enabling analysis of threshold changes whereas spontaneous behavioural change may better relate to the presence of pain and general condition of an animal [111, 112]. Performance on functional assessments, such as the rotarod test, also declines following lumbar intervertebral disc injury [46].

The assessment of pain in small animal models is imperfect but greatly increases our power to investigate the underlying pathophysiology of intervertebral disc degeneration related pain.

## 5. Conclusions

The complexity of the human intervertebral disc bares repetition. Given this inherent complexity no animal model will perfectly replicate the clinical condition. The best that can be hoped for is to mimic as closely as possible the clinical condition of degenerative disc disease. Important considerations in choosing an appropriate animal model are the absence of notochordal cells, animal and intervertebral disc size relative to humans, biomechanical forces acting upon the intervertebral disc, mechanistic concerns (i.e., mechanical injury versus chemical injury), and ethical considerations. Nonhuman primates closely match the clinical condition with regard to many of the physical and mechanistic criteria, particularly given the demonstration of spontaneous intervertebral disc degeneration in baboons and macaques. However, ethical considerations should preclude their widespread utilization.

The ovine model of disc disease possesses many desirable characteristics when considering the ideal intervertebral discs model: absence of notochordal cells, similar body mass to humans, and similar biomechanical forces acting upon the intervertebral disc. A major potential criticism of this model is the quadrupedal rather than bipedal nature of sheep. As discussed previously biomechanical studies have indicated that the ovine and human lumbar spines have good comparability in many biomechanical properties [113] in spite of the quadrupedal/bipedal dichotomy.

Certain questions will remain unanswerable in large animal models without significant advances in technology. As such, a role for small animal models will continue, particularly in the investigation of the action of specific gene products in disc degeneration through the use of genetically modified/knockout mice.

The variety of methods of inducing disc degeneration is even broader than the variation in animal models. Spontaneous models of disc degeneration, such as the chondrodystrophic dog and primate, are most likely to parallel the clinical condition in terms of underlying mechanism and time frame. However, the variability of onset and prolonged time course of the degenerative process renders such models difficult to utilize in many contexts. Investigation of regenerative therapies, for example, would be rendered exceptionally difficult if an investigator were to wait for all animals scheduled for investigation to spontaneously develop an appropriate degree of degeneration. For investigations of regenerative therapies it is thus likely that methods of inducing structural injury will be the most utilized as these enable instantaneous production of a replicable injury at a defined time point.

In conclusion no animal model will mimic the clinical condition of disc degeneration with complete fidelity. This is due to the complexity of clinical intervertebral disc degeneration and the immense influence of the subjective phenomena of pain in determining patient outcomes. Animal models will continue to play an essential role in refining our understanding of the pathophysiology of disc degeneration, developing novel therapies for this condition, and ultimately translating such therapies to the clinic.

## Figures and Tables

**Table 1:** Summary of animal models of disc degeneration.

Animal	Notochordal cells in adult intervertebral disc	Mechanism	References
Mouse	Present	*Spontaneous*	
		Knockout: Col2a1 gene/type II collagen	Sahlman et al. [18]
		Collagen IX mutation	Kimura et al. [38]
		*Mechanical*	
		Tail bending	Court et al. [39]
		Bipedal mouse	Higuchi et al. [40], Goff and Landmesser [36]
		Instability: resection of posterior elements	Miyamoto et al. [41]

Rat	Present	*Spontaneous*	
		HLA-B27 and human *β*_2_-microglobulin gene transgenic	Hammer et al. [42]
		*Mechanical*	
		Tail bending	Lindblom [43]
		Bipedal rat	Goff and Landmesser [36]
		Ilizarov-type apparatus	Iatridis et al. [44]
		Cyclical compression	Ching et al. [45]
		*Structural*	
		Stab incision	Rousseau et al. [46] and Jeong et al. [47]

Sand rat	Present	*Spontaneous*	
		Laboratory diet	Silberberg et al. [48], Gruber et al. [49], Moskowitz et al. [50]

Rabbit	Present	*Mechanical*	
		External loading device	Kroeber et al. [51]
		Adjacent segment fusion	Phillips et al. [52]
		*Structural*	
		Annulus puncture	Masuda et al. [20]
		Chemonucleolysis: chondroitinase ABC	Kiester et al. [53]
		NP aspiration	Sakai et al. [54]

Canine			
Nonchondrodystrophoid dog	Present	*Spontaneous*	Bergknut et al. [21]
		*Structural*	
		Annular injury with scalpel/drill	Keyes and Compere [55]
		Subtotal discectomy	Hohaus et al. [56]
Chondrodystrophoid dog	Absent	*Spontaneous*	Gillett et al. [57] and Bergknut et al. [21]
		*Structural*	
		Needle aspiration of NP	Serigano et al. [58]
		Chemonucleolysis: chymopapain	Melrose et al. [59]

Goat	Absent	*Structural*	
		Chondroitinase ABC	Hoogendoorn et al. [60]
		Drill bit injury/annulotomy	Zhang et al. [22]

Pig	Present	*Structural*	
		Nucleotomy	Acosta et al. [61]

Sheep	Absent	*Structural*	
		Partial thickness annulotomy	Osti et al. [62], Oehme et al. [63], Melrose et al. [64]
		Annular incision and partial nucleotomy (simulated microdiscectomy)	Oehme et al. [23]
		Chondroitinase ABC	Ghosh et al. [65]

Macaque	Present	*Spontaneous*	
		Age related degeneration	Nuckley et al. [66]

Baboon	Present	*Spontaneous*	
		Age related degeneration	Lauerman et al. [24] and Platenberg et al. [25]

Rhesus monkey	Present	*Structural*	
		Annulotomy ± collagenase	Stern and Coulson [67]
		Bleomycin injection of subchondral bone	Wei et al. [68]

NP indicates nucleus pulposus.
